# Authors’ Reply to: Is a Ratio Scale Assumption for Physician Ratings Justified? Comment on “What Patients Value in Physicians: Analyzing Drivers of Patient Satisfaction Using Physician-Rating Website Data”

**DOI:** 10.2196/21057

**Published:** 2020-10-26

**Authors:** Sonja Bidmon, Ossama Elshiewy, Ralf Terlutter, Yasemin Boztug

**Affiliations:** 1 Department of Marketing and International Management Alpen-Adria-Universitaet Klagenfurt Klagenfurt am Woerthersee Austria; 2 Department of Business Administration, Marketing and Consumer Behavior University of Goettingen Goettingen Germany

**Keywords:** online physician ratings, patient satisfaction, multiattribute models, health care management

We appreciate the comments made by Konerding [1] and are thankful for the opportunity to take part in this research dialogue. Konerding [1] states that “the zero-points for the rating scales analyzed by Bidmon et al [2] cannot be determined empirically” and “what these parameters tell us about the actual relationships between satisfaction and service attributes is unclear.” It is obvious that the arguments of Konerding [1] rely solely on the paradigm of representational theory of measurement. However, the academic literature acknowledges three theories in this domain: the representational, the operational, and the classical (we refer to Michell [3] for an excellent discussion on this topic, which has already been cited in our paper [2]). Consequently, we understand the point of view conveyed by Konerding [1], but perceive it as too narrow in the spirit of empirical research. We are pleased to clarify as follows.

First, our assumption for the scale level of the satisfaction ratings draws from a well-known conceptual framework: the 3-factor model of customer satisfaction [4-6]. This model includes the concept of linear and nonlinear relationships between satisfaction ratings of a service attribute and the overall satisfaction rating with the service (describing diminishing, constant, or increasing returns).

Second, the methodological assumptions to empirically identify the 3-factor model of customer satisfaction are clearly spelled out in our methods section (see our paper [2], subsection *Statistical Analysis*), and the approach using the log-log regression model to estimate elasticities is well established in the literature of econometric models of demand [7]. Elasticities allow the interpretation of slope coefficients in terms of diminishing, constant, and increasing returns.

Third, in our work [2], we emphasize the range and conditions where our results can be interpreted: the empirical meaning of our nonlinear slope coefficients and the prediction of changes in patient satisfaction apply to the range of publicly available ratings on the physician rating website. Explicitly assuming ratio-scale level within our specific range of satisfaction ratings is a helpful and valid assumption to empirically identify the 3-factor model of customer satisfaction (which has only been implicitly assumed in previous research). Our choice of numerical coding for the ratings (which follows common practice in empirical research) ensures that the diminishing, constant, and increasing returns are correctly identified within the relevant range of our satisfaction ratings.

Fourth, we provide a robustness check in our paper [2] to show that our empirical findings do not rely on the log-log regression model or the elasticities (this robustness check was explicitly suggested by the author of the comments [1] during the review process of our paper [2]). The most important finding from applying the alternative approach is that it leads to results that are identical to those from our main approach for the classification of the service attributes to the 3-factor model of customer satisfaction. We would like to take the opportunity to emphasize that this alternative approach (leading to the same results) should not be preferred to our main approach because it violates the principle of sparse parametrization and lacks straightforward hypothesis testing.

Taken together, we are thankful for the fruitful discussion concerning our main approach, which has led us to clearly spell out the assumptions under which our results are valid, and to emphasize the robustness check that supports the validity of our main approach. We advise researchers to bear these assumptions in mind when interpreting our results and especially when adopting our main approach to empirically identify nonlinear slopes from rating data.
